# Assessing Teacher Burnout in Elementary Education: A Study in Southwestern Greece

**DOI:** 10.1192/j.eurpsy.2025.1837

**Published:** 2025-08-26

**Authors:** P. Kallianezos, E. Ntouka, M. Bakola, A. K. Sakaretsanou, K. S. Kitsou, C. Petropoulos, S. Antonopoulou, E. Jelastopulu

**Affiliations:** 1 Department of Public Health, University of Patras, Medical School; 2 Postgraduate program of Education Management; 3 Department of Mathematics; 4 Department of Management Science & Technology, University of Patras, Patras, Greece

## Abstract

**Introduction:**

Occupational burnout in education is a major problem, especially among elementary school teachers (ESTs) who have the dual task of imparting knowledge and nurturing children’s emotional intelligence. Research in recent years focuses on understanding, preventing and addressing teachers’ burnout.

**Objectives:**

We aimed to evaluate professional burnout levels among ESTs and identify associated factors.

**Methods:**

We conducted a cross-sectional study among ESTs in southwestern Greece from September to December 2022. Participants answered to a self-administered questionnaire that included the Maslach’s Burnout Inventory Educator Survey (MBI-ES), socio-demographic, and other characteristics.

**Results:**

A total of 126 ESTs (63.5% kindergarten, 36.5% elementary school) participated in the study, 81% were female, 39.7% were 31-40 years old, and 51 (40.5%) had 11-20 years of work experience. The majority (63.5%) had a permanent job, mainly as a preschool teacher. Teachers experienced moderate to high levels of job burnout, as indicated by measures of emotional exhaustion (mean 30.03), depersonalization (mean 9.45), and feelings of low personal achievement (mean 46.42). Male teachers had higher levels of emotional exhaustion (50% vs. 46.1%) and female teachers had significantly higher rates of low personal accomplishment (80.4% vs. 66.7%). More experienced teachers showed higher emotional exhaustion (11-12 years: 58.8% vs. 6-10 years: 44.4%). Finally, kindergarten teachers were more likely to report low levels of personal accomplishment compared to other teachers (84% vs. 68.6%).

**Conclusions:**

Elementary school teachers experience moderate to high levels of job burnout, which is influenced by factors such as gender, years of service, employment status, and phase of teaching. These findings can serve as a basis for prevention strategies and interventions to reduce the effects of burnout in elementary school teachers.

**Disclosure of Interest:**

None Declared

